# Mechanical compression during repeated sustained isometric muscle contractions and hyperemic recovery in healthy young males

**DOI:** 10.1186/s40101-015-0075-1

**Published:** 2015-10-31

**Authors:** Takuya Osada, Stefan P. Mortensen, Göran Rådegran

**Affiliations:** Department of Sports Medicine for Health Promotion, Tokyo Medical University, 6-1-1, Shinjuku, Shinjuku-ku, Tokyo 160-8402 Japan; Cardiac Rehabilitation Center, Tokyo Medical University Hospital, 6-7-1, Nishishinjuku, Shinjuku-ku, Tokyo 160-0023 Japan; The Copenhagen Muscle Research Centre, Rigshospitalet, University of Copenhagen, DK-2100 Copenhagen Ø, Denmark; Department of Cardiovascular and Renal Research, University of Southern Denmark, DK-5000 Odense, Denmark; Department of Clinical Sciences Lund, Cardiology, Lund University, SE-221 85 Lund, Sweden; The Section for Heart Failure and Valvular Disease, The Heart and Lung Clinic, Skåne University Hospital, Lund, Sweden

**Keywords:** Mechanical compression, Isometric exercise, Isometric muscle contraction and relaxation, Exercising muscle blood flow, Muscle vasodilatation

## Abstract

**Background:**

An elevated intramuscular pressure during a single forearm isometric muscle contraction may restrict muscle hyperemia. However, during repeated isometric exercise, it is unclear to what extent mechanical compression and muscle vasodilatation contribute to the magnitude and time course of beat-to-beat limb hemodynamics, due to alterations in leg vascular conductance (LVC).

**Methods:**

In eight healthy male subjects, the time course of both beat-to-beat leg blood flow (LBF) and LVC in the femoral artery was determined between repeated 10-s isometric thigh muscle contractions and 10-s muscle relaxation (a duty cycle of 20 s) for steady-state 120 s at five target workloads (10, 30, 50, 70, and 90 % of maximum voluntary contraction (MVC)). The ratio of restricted LBF due to mechanical compression across workloads was determined by the formula (relaxation LBF − contraction LBF)/relaxation LBF (%).

**Results:**

The exercise protocol was performed completely by all subjects (≤50 % MVC), seven subjects (≤70 % MVC), and two subjects (≤90 % MVC). During a 10-s isometric muscle contraction, the time course in both beat-to-beat LBF and LVC displayed a fitting curve with an exponential increase (*P* < 0.001, *r*^2^ ≥ 0.956) at each workload but no significant difference in mean LBF across workloads and pre-exercise. During a 10-s muscle relaxation, the time course in both beat-to-beat LBF and LVC increased as a function of workload, followed by a linear decline (*P* < 0.001, *r*^2^ ≥ 0.889), that was workload-dependent, resulting in mean LBF increasing linearly across workloads (*P* < 0.01, *r*^2^ = 0.984). The ratio of restricted LBF can be described as a single exponential decay with an increase in workload, which has inflection point distinctions between 30 and 50 % MVC.

**Conclusions:**

In a 20-s duty cycle of steady-state repeated isometric muscle contractions, the post-contraction hyperemia (magnitude of both LBF and LVC) during muscle relaxation was in proportion to the workload, which is in agreement with previous findings. Furthermore, time-dependent beat-to-beat muscle vasodilatation was seen, but not restricted, during isometric muscle contractions through all target workloads. Additionally, the relative contribution of mechanical obstruction and vasodilatation to the hyperemia observed in the repeated isometric exercise protocol was non-linear with regard to workload. In combination with repeated isometric exercise, the findings could potentially prove to be useful indicators of circulatory adjustment by mechanical compression for muscle-related disease.

## Background

It has previously been reported that the difference in muscle blood flow in the conduit artery may be influenced by muscle mass [1, 2], vasculature [3], aging [4–7], or gender [8]. Thus, the magnitude in exercising blood flow has functioned as an indirect indicator of oxygen supply via rapid vasodilatation or increased cardiac output. Furthermore, since the interaction between voluntary muscle contractions and muscle blood flow may potentially be closely linked to exercise capacity, investigating the relationship between muscle contraction and hemodynamic response may possibly yield important information in the physical fitness or exercise training and rehabilitation fields.

Mechanical compression in voluntary isometric muscle contraction (IMC) may contribute to the transient increase in muscle blood flow following the release of restricted arterial inflow, resulting in an increase in venous return and an arteriovenous pressure gradient. In turn, local vasodilator signals contribute to exercise hyperemia with alterations in the time course of muscle blood flow magnitude during steady-state.

Representative studies demonstrated that forearm hyperemic response following IMC, measured by plethysmography, increased with isometric handgrip up to 30–40 % of maximum voluntary contraction (MVC) and was attenuated at a higher tension above 50–80 % of MVC [9, 10]. This led researchers to speculate that mechanical arterial obstruction may be dependent on the increased intramuscular pressure represented by muscle force strength [11].

Recently, an investigation of limb blood flow response has examined the role of mechanical compression due to incremental IMC [12, 13] and a single IMC [14], but there have been few reports on repeated IMC.

Another study reported that the magnitude of time-averaged brachial arterial blood flow measured by Doppler ultrasound every 10 s showed a slight, gradual increase from onset to the end of 60-s sustained IMC, ranging from 10 to 70 % of MVC but with no reduction of muscle blood flow [15].

Moreover, the magnitude of post-exercise (recovery) hyperemia immediately after the end of a single forearm sustained IMC was found to be parallel to the increase in contraction workload [8, 13] and was found to return to basal state with an exponential decay [16]. Thus, the degree of hyperemia following a brief IMC is inversely related to the degree of perfusion during IMC [9, 17].

Regarding hyperemia due to an ischemic state from exercise, a large increase in brachial blood flow is seen following the end of IMC with occluded arterial inflow, rather than IMC alone [16]. This finding may potentially indicate the acceleration of muscle blood flow after release of ischemia due to restricted and/or occluded perfusion blood flow, even from a single mechanical compression of IMC.

The abovementioned studies have focused on the relationship between elevated intramuscular pressure caused by mechanical compression of a single sustained IMC involving the “relatively small forearm flexor muscles” and “restricted comprehensive mean (averaging)-muscle blood flow and/or hyperemic recovery.”

However, it is unclear to what extent repeated muscle mechanical compression to restricted muscle blood flow and/or vasodilatation is determined by essential beat-to-beat dynamics between IMC and muscle relaxation during steady-state exercise. The hemodynamic response representing the increase in the metabolic state due to repeated mechanical vessel compression may be different from a single mechanical compression, consequently influencing the magnitude of muscle blood flow/vasodilatation between muscle IMC and muscle relaxation of the duty cycle.

Consequently, there remains a lack of information on whether the repeated IMC restricted hyperemic flow in a relaxation interval will influence the magnitude in muscle blood flow during steady-state isometric exercise, involving the relatively large thigh muscle groups of the knee extensor, using a high time-resolution ultrasound Doppler system.

Our hypothesis of steady-state repeated IMC exercise is that post-IMC hyperemia during subsequent muscle relaxation would be related to the quantity (degree) of IMC restricted muscle blood flow but not workload-dependent local signals that oppose mechanical compression of vascular beds at incremental target workloads. Therefore, the present study examined (1) if the magnitude/time course of beat-to-beat measurements for leg blood flow (LBF), blood pressure (BP), and leg vascular conductance (LVC) influences exercise hyperemia over the muscle contraction-relaxation duty cycles (20s) during repeated sustained thigh IMC of the knee extensor and (2) the role of restricted muscle blood flow during IMC with hyperemia in the subsequent muscle relaxation phase.

## Methods

### Participants

Eight healthy male volunteers aged 26 ± 2 years, height 178 ± 2 cm, and weight of 72 ± 2 kg participated in this study. The subjects were physically active, with regular recreational exercise varying from daily activities to endurance training, with no previous history of cardiovascular disease or hypertension. The study was approved by the Ethics Committee of Copenhagen and Frederiksberg, and the experiments were conducted in accordance with the guidelines of the Declaration of Helsinki. The subjects were informed of any risks and discomforts associated with the experiments before they gave their informed, written consent to participate.

### Repeated isometric knee extensor exercise

**Fig. 1:**
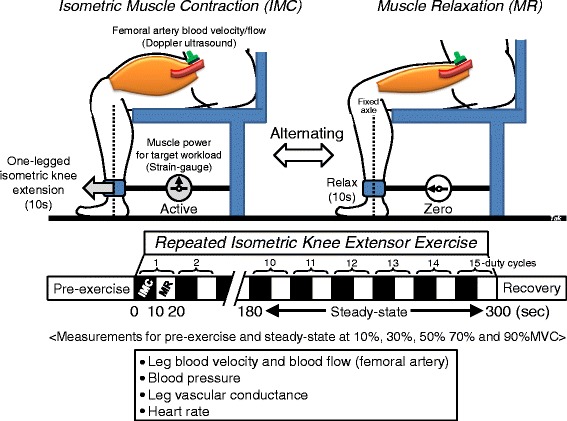
The experimental setting for repeated isometric knee extensor exercise. The isometric exercise duty cycle between 10-s isometric muscle contraction (IMC) and 10-s muscle relaxation (MR) was repeated for 5 min. Hemodynamic parameters were simultaneously measured through all experiments at 10, 30, 50, 70, and 90 % MVC, respectively. Measurement was defined at pre-exercise, as well as steady-state exercise. *%MVC* percentage of maximum voluntary contraction

The knee extensor model to determine muscle blood flow, as described in previous studies, uses solely the knee extensors of the thigh quadriceps muscle group [18]. The subject’s right thigh was positioned horizontally, with the knee joint bent in the sitting position (fixed axle in the lower leg below the knee joint). The right foot was placed into a custom-designed boot with a strain-gauge connection (Meiko Co. Ltd, Tokyo, Japan) for achievement of target workloads corresponding to varying relative values of MVC, which were displayed on a monitor. The duty cycle of muscle contraction consisted of repeated 10-s IMC–10-s muscle relaxation cycles (Fig. 1).

### Experimental protocol

Prior to the experiment, the MVC was measured by the maximum muscle contraction power throughout a single (one-legged) IMC using the right (dominant) leg, with the subject in the sitting position. The MVC was determined from the average of five repeated measures. The target workload was determined by the relative (percentage of) MVC at five different intensities (10, 30, 50, 70, and 90 % MVC, respectively). Before the exercise test, all subjects were familiarized with the exercise to maintain the target workload for repeated 10-s IMC. Following 5 min of pre-exercise, 15 repeated IMC-muscle relaxation cycles were performed at each target workload. The muscle contraction interval (10 s on/10 s off) was maintained by following an audible metronome every 10 s for the IMC and muscle relaxation phases. Measurements were performed from the 10th to 15th duty cycles at steady-state (Fig. 1). The target workload (%MVC) during sustained IMC was maintained by visual tracking of the workload, displayed in real time on a monitor connected to the strain-gauge and to the individual participants. Exercise went from 10 to 90 % MVC. The amount of time was sufficient to allow the hemodynamic parameters to return to resting control levels between 10 and 30 % MVC for 45 min and above 30 % MVC for 60 min.

The parameters (blood velocity, BP, voluntary muscle contraction power, and surface electromyography) were simultaneously recorded by beat-to-beat measurements at pre-exercise and during steady-state exercise of between 180 and 300 s of 15 repeated duty cycles at 10, 30, 50, 70, and 90 % MVC, respectively (Fig. 1).

**Fig. 2:**
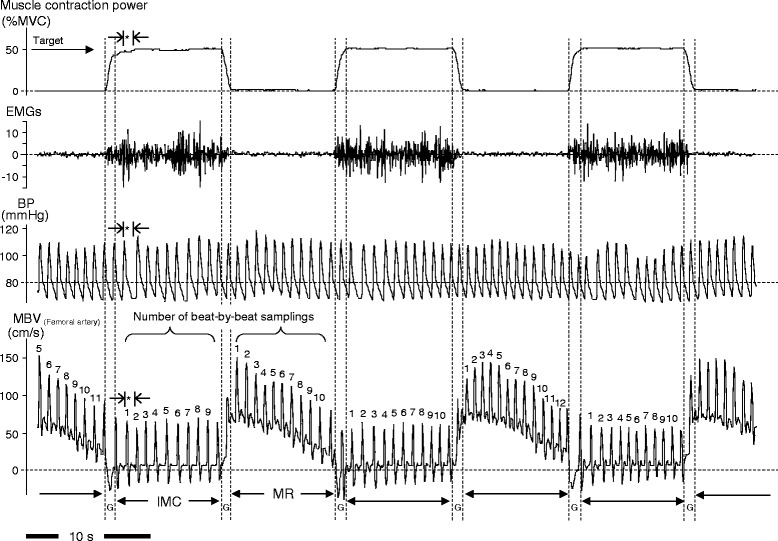
Typical magnitude of mean blood velocity, BP, EMGs, and muscle contraction power during repeated IMC and muscle relaxation at 50 % MVC. The beat-to-beat mean blood velocity (MBV) was higher in the muscle relaxation (MR) than in the isometric muscle contraction (IMC) phase due to higher muscle contraction power (intramuscular pressure) restricting blood flow perfusion during IMC. The complete consecutive beat-by-beat blood velocity profile (only in completed systole and diastole forms in →|*|←) during 10-s IMC or 10-s MR phase was measured. A fragment of the blood velocity profile in the gap (*G*) between IMC and MR was not included in the evaluations. *%MVC* percentage of maximum voluntary contraction, *EMGs* surface electromyography, *BP* blood pressure

The beat-to-beat (only in complete systole and diastole waveforms) analysis for the hemodynamic parameters was evaluated during IMC and muscle relaxation phases. A fragment of hemodynamics recording, in the gap between IMC and muscle relaxation phases, was not included in the evaluations (see Fig. 2).

### Blood velocity, vessel diameter, and calculation of leg blood flow

Measurements in the femoral artery (dominant leg) were performed using an ultrasound system (Model CFM 800; Vingmed Sound, Horten, Norway), which had previously been validated with accurate absolute values at rest and during knee extensor exercise [19–28]. The probe position was stable (<60°), and the sample volume was precisely positioned in the centre of the vessel and adjusted to cover the diameter width of the vessel.

The high temporal resolution of a time- and space-averaged and amplitude weighted-mean blood velocity profile during repeated IMC and muscle relaxation was continuously recorded by a computer using a PowerLab data acquisition system (Chart v.4.2.3 software; ADInstruments, Sydney, Australia, see Fig. 2).

The measurement instrument in the present study could not simultaneously measure both blood velocity (pulsed Doppler mode) and vessel diameter (two-dimensional mode) in the artery. Such a protocol would have required that the same experiment be performed twice: once for blood velocity and once for diameter measurement because of the difficulty in replicating both heart rate (HR) and BP responses at each target workload.

Furthermore, since detection of the entire vessel diameter change due to beat-to-beat dynamics during exercise was not simple, the preliminary measurement of the vessel diameter immediately before the end of both IMC and muscle relaxation was tested during steady-state at the five target workloads.

Preliminary measurements for the three subjects showed no significant difference (*P* = NS) in determined mean vessel diameter ((systolic vessel diameter value × 1/3) + (diastolic vessel diameter value × 2/3)) [19] across pre-exercise, immediately before the end of both 10-s IMC and 10-s muscle relaxation phases during steady-state at the five target workloads. Therefore, under perpendicular insonation at the pre-exercise (basal) state, the femoral arterial diameter measured at 9.66 ± 0.28 (range 9.0–11.2) mm, which was used to calculate LBF at rest and during repeated IMC, in accordance with accepted findings for the leg [19, 29–31], as compared to rhythmic forearm handgrip exercise with a relatively small muscle size [32].

LBF was calculated by multiplying the cross-sectional area (area = *π* × (resting vessel diameter/2)^2^) by time- and space-averaged and mean blood velocity at pre-exercise and during exercise.

### Blood pressure, heart rate, and leg vascular conductance

BP was determined using an auricular plethysmography device with oscillometric calibration, with a cuff tourniquet placed on the upper right arm (RadiaPress RBP-100, KANDS, Aichi, Japan). The HR was estimated by the beat-to-beat intervals, as per the formula: 60/beat-by-beat intervals (beat/min). The LVC was calculated as LBF divided by BP (LBF/BP) using the unit ml^/^min/mmHg.

### Muscle contraction power and surface electromyography

Muscular contraction power, measured by the strain-gauge connection, was used to confirm the subjects’ performance of isometric muscle contractions while achieving target workloads (Fig. 1). The muscle contraction power well represented the oscillations in intramuscular pressure, and a strong correlation between muscle strength and intramuscular pressure within the knee extensor muscle group during exercise has been previously demonstrated [11, 19, 20]. Thus, the fluctuations in IMC power determined by beat-to-beat analysis were measured as coefficients of variations for %MVC, which indicated the success of exercise achieved at target intensity within an acceptable range of <5 %, whether or not target workload was maintained completely during IMC. Surface electromyography activity of the vastus lateralis was additionally recorded to confirm the maintenance of muscle strength during isometric voluntary muscle contractions (Fig. 2).

### Efficiency of mechanical compression

The relative contribution of mechanical compression due to IMC on restricted LBF during muscle relaxation across workloads was evaluated by the formula (relaxation LBF – contraction LBF)/relaxation LBF (%), representing the restricted LBF ratio.

## Statistics

Data (with the exception of 90 % MVC due to the low number of subjects = 2) were analyzed using multiple analyses of variance for repeated measures and Fisher’s PLSD significant difference for post-hoc tests, when comparing more than two groups. LBF, LVC, HR, and mean BP were analyzed for the comparison among pre-exercise, IMC, and muscle relaxation (IBM SPSS statistics version 22). Statistical analysis with a linear-curve fitting regression correlation coefficient (*r*^2^), and *P* value was examined for the time course in beat-to-beat measurements of both LBF and LVC (time from initial beat on the *x* axis, both LBF and LVC on the *y* axis) during IMC and muscle relaxation phases, respectively (Microsoft Excel 2010). The correlation between mean LBF and target workload (%MVC) was examined during IMC and muscle relaxation. A *P* value <0.05 was considered statistically significant. All values are mean ± standard error.

## Results

Mean values for LBF, HR, BP, and LVC during IMC and muscle relaxation at all target workloads are shown in Table 1. Mean LBF, LVC, and HR during IMC showed no statistical change between target workloads and pre-exercise. In contrast, during muscle relaxation, there were significant increases (*P* < 0.05) in mean LBF, LVC, and HR during IMC at each target workload except 10 % MVC, compared to pre-exercise. Mean BP showed no significant increase with an increase in target workloads and was similar between IMC and muscle relaxation at all target workloads. The achieved mean %MVC was almost equal to the target workload, with acceptably low coefficients of variation (<3.5 %) representing stability during IMC at all target workloads.

**Table 1:** Mean values for LBF, HR, MBP and LVC during IMC and muscle relaxation

Measurement parameters	Pre-exercise (basal)	10-s isometric muscle contraction phase					10-s muscle relaxation phase				
		Target workload, %MVC (sub. nr.)					Target workload, %MVC (sub. nr.)				
		10 % (*n* = 8)	30 % (*n* = 8)	50 % (*n* = 8)	70 % (*n* = 7)	90 % (*n* = 2)	10 % (*n* = 8)	30 % (*n* = 8)	50 % (*n* = 8)	70 % (*n* = 7)	90 % (*n* = 2)
Achieved %MVC (%)	–	10.2 ± 0.1	29.8 ± 0.3	49.0 ± 0.4	69.9 ± 0.6	91.8 ± 3.8	–	–	–	–	–
CV for achieved %MVC (%)	–	3.0 ± 0.8	2.0 ± 0.6	2.4 ± 0.7	2.1 ± 0.3	3.1 ± 0.4	–	–	–	–	–
LBF (L/min)	0.46 ± 0.07	0.54 ± 0.06	0.61 ± 0.1	0.54 ± 0.1	0.64 ± 0.13	0.51 ± 0.22	0.85 ± 0.08	1.96 ± 0.19*^,^**	2.54 ± 0.25*^,^**	3.84 ± 0.57*^,^**	5.04 ± 2.33
HR (beat/min)	69.5 ± 3.7	70.7 ± 3.3	70.9 ± 2.8	73.2 ± 3.5	76.0 ± 3.5	75.7 ± 9.4	73.4 ± 3.4	80.0 ± 3.5*^,^**	84.9 ± 4.1*^,^**	89.0 ± 4.8*^,^**	89.5 ± 7.9
MBP (mmHg)	80.9 ± 3.2	81.2 ± 2.5	82.4 ± 2.9	80.0 ± 3.2	79.8 ± 2.4	77.2 ± 6.8	80.6 ± 2.9	81.5 ± 3.3	79.7 ± 3.2	79.3 ± 2.9	87.8 ± 7.4
LVC (mL/min/mmHg)	5.7 ± 0.9	6.7 ± 0.8	7.5 ± 1.3	6.9 ± 1.3	7.8 ± 1.4	6.5 ± 2.3	10.7 ± 1.1	24.0 ± 2.1*^,^**	32.0 ± 3.1*^,^**	47.8 ± 5.9*^,^**	55.5 ± 22.0

Significant difference (*P < 0.05); vs. pre–exercise (basal), (**P < 0.05); isometric muscle contraction vs. muscle relaxation at same target workload, exception of 90 % MVC. The “*n*” corresponds to the number of subjects (sub. nr.). The values are expressed as means ± standard error (SE)

*LBF* leg blood flow, *HR* heart rate, *MBP* mean blood pressure, *LVC* leg vascular conductance, *%MVC* percentage of maximum voluntary contraction, *CV for %MVC* coefficients of variations for %MVC via beat-to-beat

**Fig. 3:**
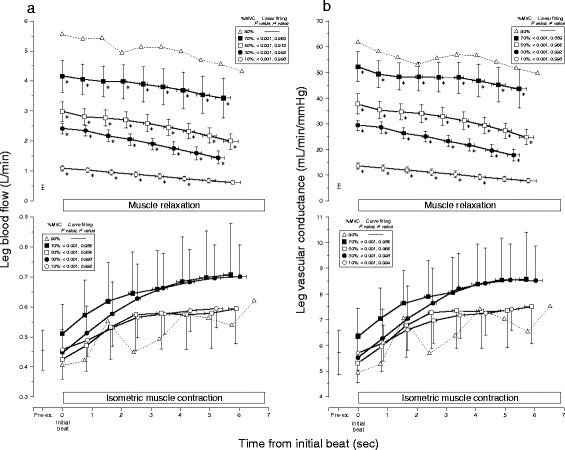
Relationship in the time course of beat-to-beat LBF (**a**) and LVC (**b**) during IMC and muscle relaxation. The magnitude of both beat-to-beat leg blood flow (LBF) and leg vascular conductance (LVC) during isometric muscle contraction (IMC) represented a statistically exponential increase (*P* < 0.001; *r* ^2^ value, range 0.956 to 0.998 for LBF, 0.966 to 0.998 for LVC) from the onset to the end of IMC at all target workloads with no significant difference among target workloads. Both beat-to-beat LBF and LVC during muscle relaxation showed a significant linear decline (*P* < 0.001; *r* ^2^ value, range 0.960 to 0.998 for LBF, 0.889 to 0.998 for LVC) from the onset to the end of muscle relaxation at all target workloads. Both beat-to-beat LBF and LVC in muscle relaxation are greater compared to pre-exercise (*P < 0.05). The regression and curve fitting for 90 % MVC were statistically weak because there were only two subjects. The values are expressed as means ± standard error (only mean value with dotted line for 90 % MVC). *Pre-ex*. pre-exercise, *%MVC* percentage of maximum voluntary contraction

Figure 3 describes the time course in beat-to-beat dynamics of both LBF and LVC. The time courses in both beat-to-beat LBF and LVC during IMC displayed an exponential (hyperbolic)-like increase (*P* < 0.001, *r*^2^ ≥ 0.956 for LBF, *r*^2^ ≥ 0.966 for LVC at 10–70 % MVC) from the onset to the end of IMC at all target workloads, although mean LBF during IMC showed no significant increase at incremental target workloads. In turn, the magnitude of the time course in both beat-to-beat LBF and LVC during IMC were similar (*P* = NS) among target workloads.

**Fig. 4:**
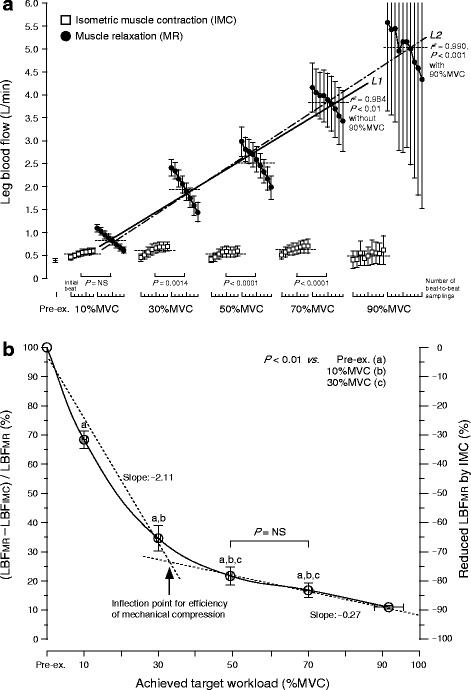
Magnitude of beat-to-beat LBF (**a**) and the efficiency of IMC mechanical compression (**b**). **a** Mean LBF (*dotted line*) during IMC showed no change among target contraction workloads, although the magnitude of beat-to-beat LBF represented a slight exponential increase from onset to the end of IMC at all target workloads. There is a close positive linear relationship between mean LBF and %MVC during muscle relaxation (*solid line*, *r* ^2^ = 0.984, *P* < 0.01 for *L1* without 90 % MVC and *dashed line*, *r* ^2^ = 0.990, *P* < 0.001 for *L2* with 90 % MVC), although the magnitude in beat-to-beat LBF represented a linear decline from onset to the end of muscle relaxation at all target workloads represented in Fig. 3. **b** The effect of IMC mechanical compression on LBF restriction in muscle relaxation is indicated. In the duty cycle of exercise, increasing mean LBF during muscle relaxation showed an exponential decay-like reduction during IMC with increasing in target workload (a *solid line*), resulting in a difference (inflection point) in mechanical restriction of LBF by IMC between 30 and 50 % MVC. The regression lines (*dotted lines*) were indicated as the formula *y* = −2.11*x* + 95.8 (*r* = −0.985) for “Pre-ex.–30 % MVC” and *y* = −0.27*x* + 35.39 (*r* = −0.998) for “50– 90 % MVC.” The slope for the efficiency of muscle mechanical compression was relatively higher under 30 % MVC than over 50 % MVC. There was a significant difference (*P* < 0.01) compared to Pre-ex. (*a*), 10 % MVC (*b*), and 30 % MVC (*c*). *LBF* leg blood flow, *Pre-ex*. pre-exercise, *NS* not significant, *%MVC* percentage of maximum voluntary contraction. The values are expressed as means ± standard error

Beat-to-beat dynamics of both LBF and LVC during muscle relaxation were significantly higher during exercise compared to pre-exercise (except at 10 % MVC for the last seven to eight beats). Consecutively, during muscle relaxation, the time course in both beat-to-beat LBF and LVC increased as a function of workload, followed by a linear decline (*P* < 0.001, *r*^2^ ≥ 0.960 for LBF, *r*^2^ ≥ 0.889 for LVC at 10–70 % MVC) from the onset to the end of muscle relaxation, at all target workloads that were workload-dependent (Fig. 3). However, mean LBF during muscle relaxation showed a positive linear increase (*P* < 0.01, *r*^2^ = 0.984 without 90 % MVC, *P* < 0.001, *r*^2^ = 0.990 with 90 % MVC) at incremental target workloads (in Fig. 4a, the dotted line shows mean LBF and the double dashed/solid line is the regression line).

The effects of mechanical compression on LBF over the duty cycle are shown in Fig. 4b. The difference in LBF magnitude between IMC and muscle relaxation revealed an increasing mean LBF during muscle relaxation as a function of the exponential decay-like reduction in muscle relaxation LBF, due to mechanical obstruction by IMC with an increase in target workloads. This pattern resulted in a difference (inflection point) in efficiency of restricted LBF by mechanical compression/obstruction between 30 and 50 % MVC (see ↑ in Fig. 4b). The regression line is represented as *y* = −2.11*x* + 95.8 (*r* = −0.985) for “pre-exercise–30 % MVC” and *y* = −0.27*x* + 35.39 (*r* = −0.998) for “50–90 % MVC.” The slope for the efficiency of mechanical compression was relatively higher below 30 % MVC (a steeper slope) than above 50 % MVC (a milder slope).

**Fig. 5:**
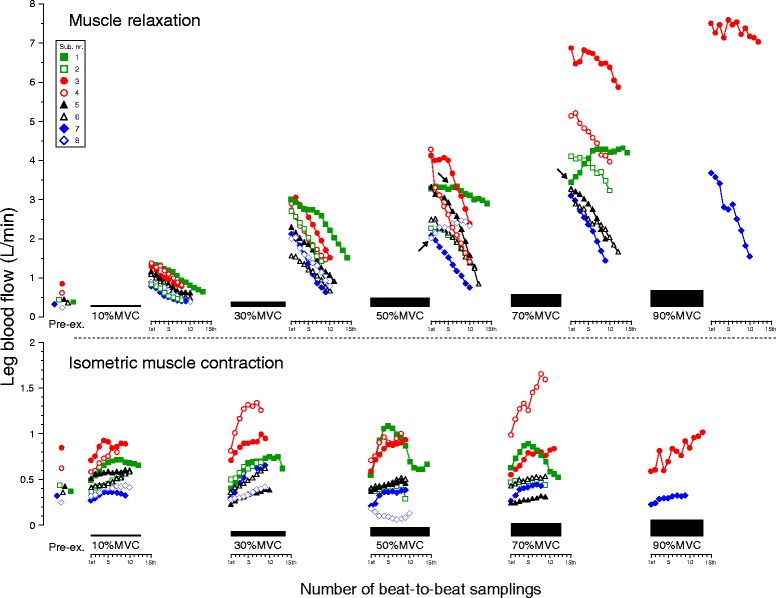
Magnitude of individual beat-to-beat LBF. During isometric muscle contraction with stable target workloads (coefficients of variations <3.1 %), the magnitude of beat-to-beat leg blood flows (LBF) displayed various patterns such as steep (*filled circle* and *empty circle*), gradual (*filled triangle*, *empty triangle*, *empty square*, and *filled diamond*), and biphasic (*filled square*). A decrease in magnitude was shown in one subject (*empty diamond*) at 50 % MVC. Most of the individual LBF magnitudes were described as an exponential decay (steep decrease) during muscle relaxation at all target workloads except for one subject (*filled square*) at 70 % MVC. The explanation of arrows is indicated in the text. *Pre-ex*. pre-exercise, *%MVC* percentage of maximum voluntary contraction

**Fig. 6:**
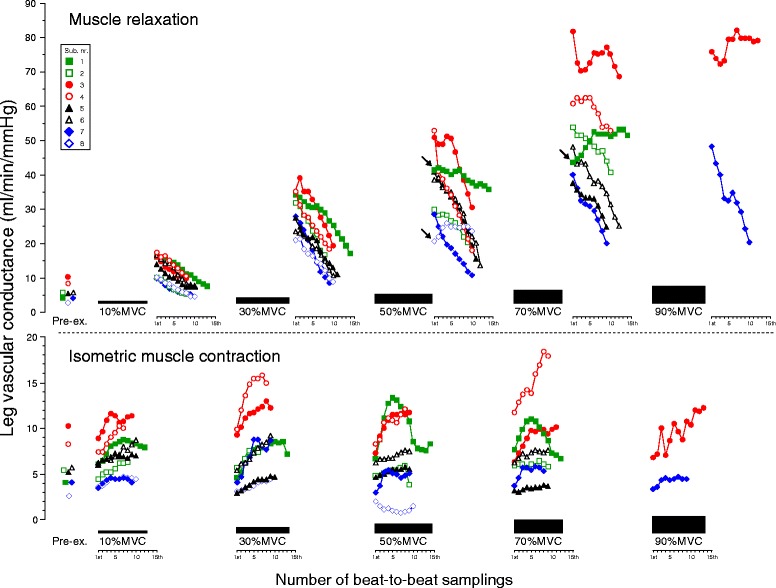
Magnitude of individual beat-to-beat LVC. The pattern of individual leg vascular conductance (LVC) was similar to that of leg blood flow, since blood pressure showed no significant difference between isometric muscle contraction and muscle relaxation. The range of values in muscle relaxation was greater than the isometric muscle contraction phase, indicating enhanced vasodilatation (hyperemic response) in muscle relaxation. The explanation of arrows is indicated in the text. *Pre-ex*. pre-exercise, *%MVC* percentage of maximum voluntary contraction

**Fig. 7:**
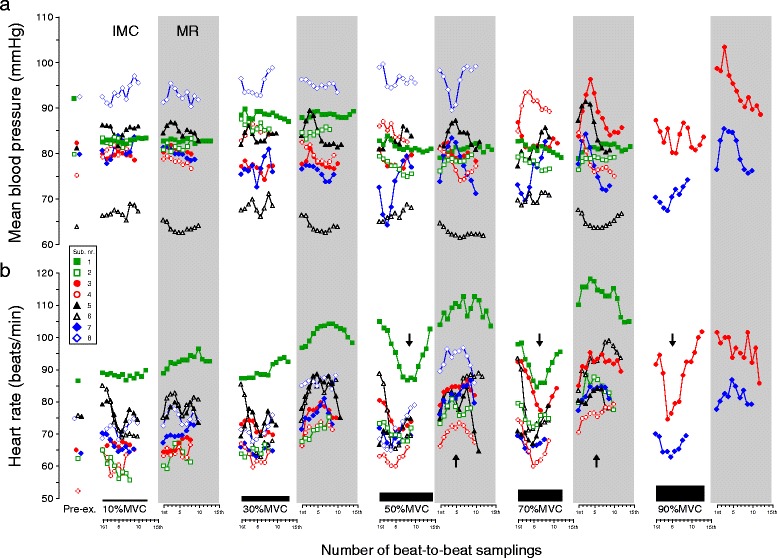
Magnitude of individual beat-to-beat (**a**) mean blood pressure and (**b**) heart rate. Variability in mean blood pressure and heart rate in individual subjects. The explanation of arrows is indicated in the text. *IMC* isometric muscle contraction, *MR* muscle relaxation, *Pre-ex*. pre-exercise, *%MVC* percentage of maximum voluntary contraction

The magnitudes of beat-to-beat hemodynamics (LBF, LVC, BP, and HR) during repeated IMC-muscle relaxation among subjects were variable, though the intra-subject variations were minor among workloads (see Figs. 5, 6, and 7 and the legends). During muscle relaxation, the beat-to-beat magnitude of both LBF and LVC declined linearly from onset to the end of muscle relaxation phase, with low variation among subjects. However, during the IMC phase, most subjects showed a gradual increase in both LBF and LVC, with an exponential (hyperbolic)-like pattern (Figs. 5 and 6). Beat-to-beat variability in BP and HR in individual subjects was shown in Fig. 7.

## Discussion

The present study demonstrated that LBF at steady-state in the femoral arteries of the predominant limb changed during a series of isometric, submaximal IMC interspersed with muscle relaxation in healthy young males. The duty cycle amounted to 20 s, with 10 s of isometric contraction and 10 s of full relaxation. The majority of the results indicated that during IMC, despite the mechanical hindrance to LBF, a clear vasodilatation occurs. In addition, this study shed new light on the relative contribution of mechanical obstruction and vasodilatation to the muscle contraction induced-hyperemia observed during repeated isometric exercise, which may potentially be a key for blood flow (oxygen) supply via leg muscle compression. These findings are discussed in the following section.

### Magnitude or dynamics of the time course in beat-to-beat vasodilatation

The present study clearly demonstrated that the time course of both beat-to-beat LBF and LVC displayed activity of vasodilatation during repeated IMC exercise but not in a single IMC. Changes in LBF through the time course of beat-to-beat dynamics during 10-s IMC showed a slightly exponential increase of approximately 200 ml/min (which was similar between different workloads) and which resulted in no observed reduction of LBF during IMC, as a function of mechanical arterial obstruction at incremental workloads (Fig. 3).

Despite similar substantial constraints on muscle blood inflow by mechanical compression of the vasculature during IMC with incremental target workloads, workload-dependent vasodilatation is clearly observed in beat-to-beat LBF and LVC during muscle relaxation (Fig. 3), which parallels the magnitude of hyperemic recovery response (Fig. 4a). Consequently, signals mediating vasodilatation and hyperemia during muscle relaxation also contribute to the increase in LBF at all workloads, even repeated IMC (Fig. 4a). This is in partial agreement with previous studies reporting on forearm blood flow and vascular conductance after the end of brief isometric handgrip exercise for 6 s [8] and 1 s [7].

The rate of decline of the beat-to-beat LBF (the slope of the regression line) over the time course during muscle relaxation was in the same range for the five target contraction workloads, with no relation to the target workload (Fig. 3). This suggests that both of the LBF and LVC attenuation ratios during 10-s muscle relaxation from peak vasodilatation immediately at the end of IMC across workloads represent a similar time course (e.g., rate of return to basal condition) regulated by metabolic factors, such as accumulated vasodilator substances dependent on the workload [33].

The magnitude in LBF during (repeated) 10-s IMC following 10-s muscle relaxation in the present study is partially in agreement with a previous study reporting that averaged brachial arterial blood flow (every 10 s) measured by Doppler ultrasound during a 60-s sustained isometric forearm muscle contraction (handgrip exercise) gradually increased to an approximately similar level (between 137 and 160 ml/min), compared to a pre-exercise level of 87 ml/min between 10 and 70 % MVC workloads [15]. This may suggest that the pattern of magnitude in vasodilatation during sustained IMC is similar between forearm and leg.

There is still a lack of information about the time course in beat-to-beat muscle blood flow during IMC, and furthermore, the discrepancy in beat-to-beat dynamics (achieved absolute value) between forearm and leg exercise may be due to variations in the exercise model (single sustained/repeated IMC or contraction intervals) or the contraction manner between forearm finger flexors and thigh and knee extensor muscles. The hemodynamics in the femoral artery during thigh IMC does not completely exclude the influence of lower leg activation because there is no cuff occlusion of lower blood inflow.

Oxygen availability influences muscle energy metabolism through the role of muscle blood flow in exercise metabolism and muscle fatigue. Some previous studies have reported that the limitation in LBF during IMC may influence the attenuation of voluntary muscle strength through muscle fatigue, via accumulation of metabolic products [13, 34–37]. However, muscle strength in the present study was achieved stably at target workloads (%MVC), with coefficients of variation for %MVC <3.1 % during IMC across target workloads (Table 1). Therefore, the shorter interval (10 s) of sustained IMC may not represent muscle fatigue during steady-state 2 min, resulting in no potential limitations of LBF for oxygen delivery in any workloads sessions, except 90 % MVC.

In the present study of a non-pharmacological intervention, the regulation of beat-to-beat dynamics between IMC and muscle relaxation is still unclear. Particularly, the time course in beat-to-beat muscle vasodilatation may be influenced by the external factors of exercise intensity (workload), exercise frequency (contraction duty cycle), and exercise time (non-/steady-state or exhausted state); however, the specific exercise model used may provide new insight into the regulation of vasodilatation during repeated IMC.

The reasons for vasodilatation during sustained muscle contraction, as well as post-contraction hyperemia, could be explained by the interaction between mechanical compression power/strength and vasodilatation [13]. During muscle contraction, muscle blood flow is increased by local vasodilatation due to vasoactive substances; however, the response is opposed by mechanical muscle forces that decrease muscle blood inflow during muscle contraction. Immediately post-contraction, these two factors (vasodilatation by vasoactive metabolite substance and mechanical compression) both work to increase muscle blood flow, and hyperemic response is markedly observed.

In the present study, a possible explanation for an exponential increase in both beat-to-beat LBF and LVC during the 10 s IMC phase may be related to the vasoactive substances released with muscle contractions, such as nitric oxide, adenosine, and acetylcholine [38, 39]. Furthermore, since a similar magnitude in the time course for beat-to-beat dynamics (LVC and LBF) was observed with an exponential increase (from onset to the end of the contraction) across workloads (Fig. 3), the work in vasodilatation via vasoactive substances released with muscle contractions may show a gradual increase against stable muscle power/strength activity over time.

There is regulation of vasodilatation in microcirculation, despite mechanical obstruction of muscle blood flow. Rapid vasodilatation during each cardiac cycle may contribute to evenly maintain LBF during IMC, without affecting the reflex of sympathetically mediated signals for vasoconstriction, which may have no effect on functional sympatholysis [40–43] and/or muscle metaboreflex [44]. Moreover, this pattern likely involves the coordination of metabolic signals for vasodilatation within muscle fibers of varying levels of activation and the distribution of flow corresponding to intramuscular pressures and gradients, including variations in muscle depth [45, 46].

### Effect of mechanical compression on muscle blood flow and vasodilatation

Another finding of our study was that the restricted muscle relaxation LBF due to IMC by mechanical compression during a duty cycle of muscle contraction and relaxation may not be dependent on workload. There is a non-linear relationship between the IMC restricted muscle relaxation LBF ratio and workload (Fig. 4b).

As a result, the increasing LBF during muscle relaxation revealed an exponential decay-like reduction induced by IMC with an increase in target workloads. The slope of the restricted LBF ratio (corresponding to efficiency of mechanical compression) was steeper in 30–50 % MVC than in 50–90 % MVC, resulting in a difference (inflection point) in efficiency of restricted LBF by mechanical compression (see the arrow in Fig. 4b). Consequently, this finding may potentially indicate that mechanical compression-related vasodilatation may partially support isometric muscle contractile accumulated metabolite-induced vasodilatation under 30 % MVC less than over 50 % MVC. Furthermore, this finding may be linked with previous findings which showed that the exercise pressor reflex did not modulate vasodilatation in the exercising leg during low workloads (below 30 % MVC) [44, 47, 48].

The present data suggest that the relative contribution of mechanical compression restricted LBF was large in lower workloads, and LBF increased linearly with IMC workloads, which is in agreement with previous findings, which show a tight coupling of muscle blood flow to metabolism, such that muscle blood flow increases with exercise intensity to meet the oxygen demands of the working muscle [20].

It is speculated that the relative contribution of mechanical compression restricted LBF may be different between low and high workloads, which may influence the magnitude in increasing venous return, HR, and cardiac output.

Previous studies have reported that during forearm isomeric exercise (handgrip) at lower workloads of up to 20 % MVC [49] or 30 % MVC [10], forearm blood flow (measured by plethysmography) increased, whereas at higher workloads of 50–80 % MVC, it decreased towards [9] or below [49, 50] the resting state. This finding may suggest that the degree of hyperemia following a brief IMC is inversely related to the degree of perfusion during IMC [9, 17]. Thus, these previous investigations suggest that the role of mechanical compression may be linearly related to contraction workload during a single sustained IMC. However, in the present study, during repeated IMC, the non-linear relationship between the IMC restricted muscle relaxation LBF ratio and workload may represent essential mechanical work for muscle blood flow regulation. This discrepancy may be due to the relationship between femoral artery blood flow for knee extensor quadriceps muscle group and brachial artery blood flow for forearm finger flexor muscle group.

### Alterations in beat-to-beat heart rate and blood pressure in contraction duty cycle

The present study showed that mean HR was significantly (*P* < 0.05) higher during muscle relaxation phase than the IMC phase at each target workload, while the mean BP showed no significant change (Table 1).

During IMC, an increase in HR following isometric exercise may potentially be due to some mechanism in the exercise pressor reflex elicited by passive or external muscle compression, suggesting that, like the classical myogenic response, it is at least partly mediated by a mechanosensitive mechanism [51–56] and/or baroreflex regulation [57, 58].

However, in the present study, the mechanism causing the increase in HR during muscle relaxation but not during sustained IMC is still unknown. A possible explanation for this is that the steep increase of venous return (with release of arteriovenous pressure gradient) forwarded to the heart from the lower leg immediately after the end of IMC may cause an increased stroke volume (Frank-Starling effect) and HR (Bainbridge reflex).

The time course in beat-to-beat HR was not parallel to either beat-to-beat LBF or LVC dynamics during muscle relaxation (Figs. 5, 6, and 7), which showed a negative linear correlation (steep decrease) for beat-to-beat dynamics at all target workloads (with the exception of two subjects at 50 % MVC and one subject at 70 % MVC, see the arrow in Figs. 5 and 6) with fewer individual variations. This may indicate that muscle metabolite-induced local beat-to-beat vasodilatation contributing to increasing beat-to-beat LBF may not be closely related to beat-to-beat HR (increasing in stroke volume).

Interestingly, the fluctuation in both beat-to-beat LBF and LVC for some subjects had an inflection point around the fifth to sixth beat after onset of IMC. It is speculated that the individual variations in vasodilatory response may be related to the regional differences in muscle contractile patterns, muscle fiber orientation, thickness, curvature of the muscle fibers, and spatial-temporal heterogeneity of vasodilatation accompanying voluntary muscle contraction force [58].

In a previous study, sustained isometric forearm handgrip exercise leads to an increase in both BP and HR at higher intensities [15], which may have been largely influenced by a balance between neural vasoconstriction and elevated forearm perfusion pressure to counteract increased intramuscular pressure [55].

However, there was less change in BP during the present exercise periods (Table 1), especially during the highest levels of exercise and immediately after the end of IMC when the muscle blood flow to one leg suddenly increased with 4–5 L/min (Fig. 3). Therefore, in a repeated isometric exercise model of duty cycles (shorter 10-s IMC on/10-s relaxation off), it is speculated that increasing LBF with vasodilatation (increasing LVC) during muscle relaxation may be compensated by the increasing net-HR, as well as cardiac output, without increase in BP related to IMC (shorter duration of sustained isometric muscle strength) as there is less activity of reflex control.

A defined breathing pattern during exercise was not strictly controlled in the present study. This may influence the hemodynamics between IMC and muscle relaxation via, for instance, potential breath-holding at higher IMC workloads. During IMC, there was some indirect evidence to suggest a respiratory influence on HR similar to that observed in the Valsalva maneuver, mediated by reflex sympathetic activity [59]. This was observed in most subjects as an inflection point around the fifth to sixth heart beat after onset of IMC as well as muscle relaxation above 50 % MVC (see the arrow in Fig. 7).

Furthermore, the steep increase in BP at the onset of the muscle relaxation phase observed in some subjects might be caused by a rapid increase in venous return, due to normalized intrathoracic pressure, which may be induced. Since these intra-subject variations, including the point of inflection in HR as well as BP, may be small across workloads (Fig. 7), circulatory and reflex control may be influenced by inter-individual variation in the areas of active muscle mass, fiber type, or breathing pattern.

### Perspective, limitations, and methodological considerations

If a mechanical obstruction influences vasodilatation of recovery hyperemia, using the repeated IMC exercise with the duty cycle (10-s on/10-s off) protocol, a non-linear relationship with regard to workload may well represent the interaction among restricted blood flow of muscle mechanical compression and increasing blood flow magnitude of metabolic vasodilation during incremental exercise workloads. In healthy young males, it would be logical that during steady-state repeated exercise, the relative contribution of muscle contraction restricted blood flow may be effective during relatively low workloads. Since the present findings may suggest that muscle contraction power is related to the magnitude of vasodilatation, this may potentially be a useful clinical indicator for the mechanical effect following (1) alterations in muscle mass/volume states such as musculoskeletal disorders, locomotorium disease or disuse syndrome with or without resistance or physical training and related to orthopedic surgery or rehabilitation and (2) alterations in intrinsic-extrinsic circulatory adjustment due to changes in body-limb position such as orthostatic disturbance.

#### Sample size and exercise protocol

The sample size (eight healthy young males) is insufficient for conclusive evidence regarding the difference in age or gender of repeated IMC’s mechanical compression to vasodilatation. In addition, since only two subjects were able to completely perform at the maximum workload of 90 % MVC, no robust conclusions can be demonstrated by a statistical power analysis. However, since the role of mechanical compression was not significantly different between 50 and 70 % MVC (in seven subjects) as seen in Fig. 4b, a similar relationship (a single exponential decay with an increase in workload) was determined in the range below 70 % MVC because of inflection point distinctions between 30 and 50 % MVC.

The time (interval) between IMC and muscle relaxation phases may be a major factor in exercise duration to exhaustion. In the present study, even though the 10-s muscle relaxation recovery phase against fatigue began after 10-s IMC, six subjects were not able to achieve steady-state at 90 % MVC.

In contrast, a shorter duration (for instance, under 5 s) of IMC (higher muscle contraction frequency) may not be suitable for the evaluation of consecutive beat-to-beat dynamics between IMC and muscle relaxation; moreover, this may suggest that rapid mechanical compression is likely in rhythmic/isotonic muscle contraction exercise. However, a longer duration (e.g., over 15 s) of IMC may induce a neurological response via activity of reflex control, compared to the present exercise protocol.

#### Respiratory manner

In the present study, respiratory control (regulation of the breathing cycle during the IMC-muscle relaxation phases) was not strictly performed. This may potentially influence the difference in hemodynamics between IMC and muscle relaxation due to potential breath-holding via alterations in venous return and/or pulmonary stretch receptor activity.

#### Vessel diameter during duty cycle

Since a real-time vessel diameter change via cardiac cycle (systole and diastole) was not detected in the present study, a slight over/under-estimation of both LBF and LVC may have been included.

#### Samplings for beat-to-beat analysis during IMC and muscle relaxation

It is unclear how the fragmentation of LBF components (one less cardiac beat) in the gap between the IMC and muscle relaxation phases may influence the magnitude in peak hyperemia with initial and consecutive beats, at onset of IMC and muscle relaxation (see →|*|← in Fig. 2). However, blood velocity in a single beat in the gap between the IMC and muscle relaxation may have less influence on the initial beat of beat-to-beat dynamics.

Since the number of heart beats was dissimilar in IMC phases and muscle relaxation phases, the mean value for beat-by-beat hemodynamics was estimated by the minimum sampling number. Thus, the expressed fitting curve may not completely cover the individual hemodynamics patterns because the last few beats were excluded. However, these excluded beats may have less influence on the statistical fit expressing the dynamics of the time course in beat-by-beat measurements because of the achievement of steady-state.

## Conclusions

This study examined alterations in the time course (magnitude/dynamics) of leg beat-to-beat vasodilatation (LBF and LVC) over repeated thigh IMC-muscle relaxation, over a wide range of isometric muscle contractions of the thigh from 10–90 % MVC. The findings revealed a symmetry of metabolic-induced muscle vasodilatation during both the IMC and muscle relaxation phases of the isometric duty cycle, reflecting intensity-dependent signals that oppose mechanical compression of vascular beds. Mainly, the exponential (hyperbolic)-like increase in both beat-to-beat LBF and LVC during IMC across all workloads may indicate an underlying regulation of vasodilatation in microcirculation, despite the mechanical obstruction of bulk flow. Additionally, mechanical compression due to repeated IMC that restricted hyperemic blood flow may potentially be effective during relatively low workloads.

## Abbreviations

BP: blood pressure
CV: coefficients of variations
EMGs: surface electromyography
HR: heart rate
IMC: isometric muscle contraction
LBF: leg blood flow
LVC: leg vascular conductance
MBV: mean blood velocity
MR: muscle relaxation
MVC: maximum voluntary contraction
SE: standard error
